# Inhibitory Interactions of *Aspalathus linearis* (Rooibos) Extracts and Compounds, Aspalathin and *Z*-2-(β-d-Glucopyranosyloxy)-3-phenylpropenoic Acid, on Cytochromes Metabolizing Hypoglycemic and Hypolipidemic Drugs

**DOI:** 10.3390/molecules21111515

**Published:** 2016-11-12

**Authors:** Oelfah Patel, Christo Muller, Elizabeth Joubert, Johan Louw, Bernd Rosenkranz, Charles Awortwe

**Affiliations:** 1 Biomedical Research and Innovation Platform, South African Medical Research Council, P.O. Box 19070, Tygerberg 7505, South Africa; oelfah.patel@mrc.ac.za (O.P.); johan.louw@mrc.ac.za (J.L.); charles.awortwe@mrc.ac.za (C.A.); 2 Post-Harvest and Wine Technology Division, Agricultural Research Council, Infruitec-Nietvoorbij, Private Bag X5026, Stellenbosch 7599, South Africa; JoubertL@arc.agric.za; 3 Department of Food Science, Stellenbosch University, Private Bag X1, Matieland 7602, South Africa; 4 Division of Clinical Pharmacology, Department of Medicine, Faculty of Medicine and Health Sciences, University of Stellenbosch, P.O. Box 241, Cape Town 8000, South Africa; rosenkranz@sun.ac.za

**Keywords:** rooibos, aspalathin, *Z*-2-(β-d-glucopyranosyloxy)-3-phenylpropenoic acid, herb-drug interaction, CYP2C8, CYP2C9 and CYP3A4

## Abstract

Rooibos extract, due to its glucose and lipid lowering effects, has potential as a nutraceutical for improvement of metabolic dysfunction. Potential herb-drug interactions as a result of the use of natural products are of increasing concern. Cytochrome P450 enzymes, CYP2C8, CYP2C9, and CYP3A4, are important in the metabolism of hypoglycemic drugs, such as thiazolidinediones (TZDs) and sulfonylureas, and hypocholesterolemic drugs, such as atorvastatin. This study investigated the effects of rooibos extracts, prepared from “unfermented” and “fermented” rooibos plant material and two of the major bioactive compounds, *Z*-2-(β-d-glucopyranosyloxy)-3-phenylpropenoic acid (PPAG) and aspalathin (ASP), on Vivid^®^ recombinant CYP450 enzymes. Unfermented (GRT) and fermented (FRE) rooibos extracts inhibited the activity of CYP2C8 (7.69 ± 8.85 µg/mL and 8.93 ± 8.88 µg/mL, respectively) and CYP3A4 (31.33 ± 4.69 µg/mL and 51.44 ± 4.31 µg/mL, respectively) based on their respective IC_50_ concentrations. Both extracts dose- and time-dependently inhibited CYP2C8 activity, but only time-dependently inhibited CYP2C9. CYP3A4 showed concentration-dependent inhibition by ASP, GRT, and FRE at 25, 50, and 100 µg/mL concentrations. ASP, GRT, and FRE time-dependently inhibited CYP3A4 activity with GRT and FRE showing a more potent time-dependent inhibition, comparable to erythromycin. These findings suggest that herb-drug interactions may occur when nutraceuticals containing rooibos extracts are co-administered with hypoglycemic drugs such as TZDs, sulfonylureas, and dyslipidemic drug, atorvastatin.

## 1. Introduction

*Aspalathus linearis* (Burm.f.) Dahlg. (Fabaceae), a legume commonly referred to as rooibos, is a member of the fynbos biome native to the Western Cape region of South Africa. The plant is processed to produce “unfermented” (green; unoxidised) and “fermented” (oxidised) rooibos, mainly for consumption as herbal tea [1]. Studies on the health benefits of fermented rooibos tea have confirmed that it alleviates oxidative stress [2], and has anti-mutagenic [3], anti-cancer [4,5,6], and anti-inflammatory [2] effects. Furthermore, rooibos extracts have been demonstrated to improve insulin resistance and related metabolic disturbances [7,8]. The anti-diabetic [9,10], anti-obesity [11], and cardio-protective effects [12,13,14] of rooibos extracts are of specific relevance given the global increase in the prevalence of diabetes and obesity [15,16,17]. These health promoting effects of rooibos have been attributed to its flavonoids including aspalathin, isoorientin, orientin, rutin, and nothofagin, as well as the phenylpropenoid glucoside, *Z*-2-(β-d-glucopyranosyloxy)-3-phenylpropenoic acid (PPAG). Aspalathin, a *C*-glucosyl dihydrochalcone, unique to rooibos, has anti-diabetic potential [18,19] and protects cardiomyocytes in the diabetic heart by increasing glucose oxidation and modulating fatty acid utilization [20]. Both aspalathin and nothofagin were shown to improve diabetic vascular inflammatory disease [21]. PPAG displayed its anti-diabetic effect by increasing in vitro glucose uptake and improving glucose tolerance in obese Wistar rats [22], as well as protecting pancreatic beta cell mass [23,24]. The protective effect of PPAG in combination with metformin against high glucose-induced cell apoptosis has been demonstrated in H9c2 cardiomyocytes [25].

The use of natural products to improve general health or to treat a range of conditions has escalated in both developed and developing countries [26,27]. Natural products are perceived to be safer and without unwanted side effects attributed to conventional medicines. This perception is greatly based on anecdotal evidence often without scientific verification. Regardless, many plant-based nutraceuticals, including those derived from rooibos, are currently being developed or are already in use. The safety and efficacy of such nutraceuticals as supplements and adjunctive therapies to chronic medications, specifically those used to treat metabolic disorders, such as type 2 diabetes and hyperlipidemia, have often not been established. The adjunctive use of natural products with chronic medications for these metabolic disorders could potentially induce adverse herb-drug interactions.

Cytochrome P450 (CYP) enzymes are mainly responsible for the metabolism of drugs and other compounds including phytochemicals in the liver, kidney and intestines. Approximately 80% of conventional drugs are metabolized by specific enzymes (CYP1A2, CYP2C9, CYP2C19, CYP2D6, CYP2E1, and CYP3A4) belonging to subfamilies, CYP1, CYP2, and CYP3 [28,29]. Metformin, a first-line anti-diabetic drug, is metabolized by CYP2C11, CYP2D1, and CYP3A1/2, while glyburide and pioglitazone, other known hypoglycemic drugs, are metabolized by CYP2C9, CYP3A4, and CYP2C8. Atorvastatin used to treat hypercholesterolemia is metabolized by CYP3A4. Inhibition of these specific CYPs by phytochemicals can affect the pharmacodynamics of these drugs, leading to toxicity or, alternatively, reduced efficacy [30]. When two substrates (phytochemicals and drugs) compete for the same receptor site, the more potent inhibitor will exert control over the weaker inhibitor, thus resulting in decreased metabolism of the respective substrate and, in the case where the drug is the weaker inhibitor, alter its pharmacodynamic properties [30]. Most drugs and xenobiotics including dietary polyphenols have the ability to bind to CYP3A4 as substrates. The chemical structure of polyphenols and, specifically, their functional groups play an important role in their metabolism. Flavonoids, common in the diet, are responsible for the modulation of the clinically relevant CYP2C8, CYP2C9, and CYP3A4 enzymes [31]. This modulation can alter drug metabolism through changes in the expression or activity of CYP enzymes, thereby affecting the plasma concentration of co-administered chronic medications [31].

The purpose of this study was, therefore, to investigate the inhibitory effects of polyphenol-enriched unfermented rooibos extract (GRT), and fermented rooibos extract (FRE), (*Z*-2-(β-d-glucopyranosyloxy)-3-phenylpropenoic acid (PPAG) and aspalathin (ASP) on Vivid^®^ recombinant CYP450 enzymes CYP2C8, CYP2C9, and CYP3A4. FRE is the same extract previously used by Mazibuko et al. [8] and Dludla et al. [14] to demonstrate increased basal and insulin-stimulated glucose uptake in C2C12 skeletal muscle cells and cardiomyocytes, respectively.

## 2. Results

### 2.1. PPAG and Flavonoid Content of Extracts

**Figure 1:**
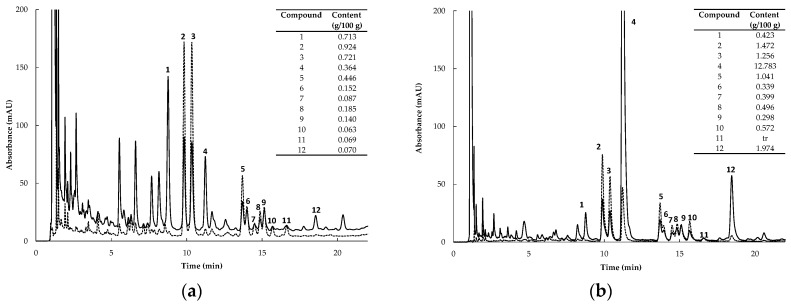
HPLC chromatograms of (**a**) FRE (fermented rooibos extract) and (**b**) GRT (unfermented rooibos extract) at 288 and 350 nm (solid and dotted lines, respectively). Content values of compounds are expressed as g/100 g extract (**1**, PPAG (*Z*-2-(β-D-glucopyranosyloxy)-3-phenylpropenoic acid), **2**, isoorientin; **3**, orientin; **4**, ASP (aspalathin); **5**, quercetin-3-*O*-robinobioside; **6**, vitexin; **7**, hyperoside; **8**, rutin; **9**, isovitexin; **10**, isoquercetrin; **11**, luteolin-7-*O*-glucoside, and **12**, nothofagin).

High Performance Liquid Chromatography (HPLC) chromatograms of the extracts are depicted in Figure 1. Content values for the individual compounds of the extracts are embedded in the respective chromatograms. GRT contained substantially higher levels of flavonoids than FRE, largely due to its high aspalathin content. It was the major compound in GRT, comprising ca. 12% of the extract, compared to 0.36% of FRE. Compounds present in GRT at >1% were nothofagin, orientin, isoorientin, and quercetin-3-*O*-robinobioside. The PPAG content of GRT and FRE were 0.42% and 0.71%, respectively.

### 2.2. Qualitative Screening of Extracts and Compounds

**Figure 2:**
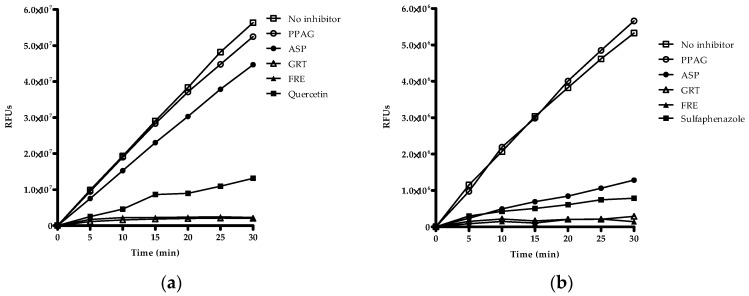

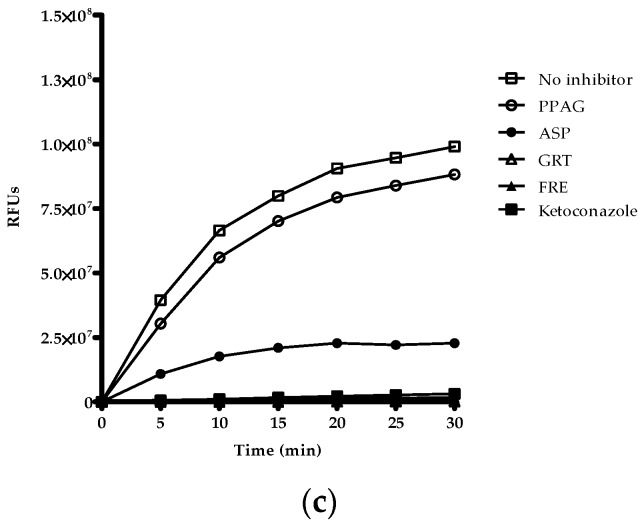
Qualitative screening of drugs, compounds and extracts based on their inhibitory potency of Vivid^®^ CYP2C8, CYP2C9, and CYP3A4 enzymes. Inhibitory activity of PPAG (*Z*-2-(β-d-glucopyranosyloxy)-3-phenylpropenoic acid), ASP (aspalathin), GRT (unfermented rooibos extract), and FRE (fermented rooibos extract) at 100 µg/mL for (**a**) CYP2C8, and (**b**) CYP2C9, and 200 µg/mL for (**c**) CYP3A4. The test samples were co-incubated with NADPH and substrate for 30 min. Quercetin, sulfaphenazole, and ketoconazole at 10 µM were included as positive inhibitors. Data are presented as RFU (relative fluorescence units) for two independent assays done in duplicate (*n* = 4).

Qualitative screening of extracts and compounds was used to identify potential inhibitory effects on CYP2C8, CYP2C9 and CYP3A4. The organic solvents such as methanol, acetonitrile and DMSO showed no inhibitory effects on the respective enzymes (Supplementary Materials, Figure S1a–c). Quercetin, sulfaphenazole, and ketoconazole were the selected positive inhibitors for CYP2C8, CYP2C9, and CYP3A4, respectively. GRT and FRE inhibited the reaction rate of CYP2C8, CYP2C9, and CYP3A4 as measured over 30 min (Figure 2a–c). ASP showed 24% inhibition of CYP2C9 at 100 µg/mL and 23% inhibition of CYP3A4 at 200 µg/mL activity, while PPAG had no effect on any of these enzymes.

### 2.3. IC_50_ Determination

**Figure 3:**
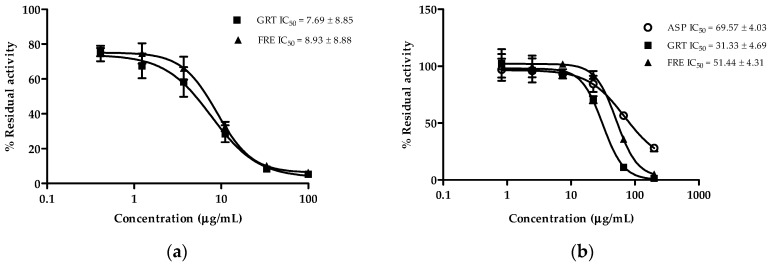
Percentage remaining activity of (**a**) CYP2C8 and (**b**) CYP3A4 after 30 min co-incubation with ASP (aspalathin), GRT (unfermented rooibos extract) and FRE (fermented rooibos extract) with NADPH and substrates. Data are the average values of two independent assays done in duplicate (*n* = 4).

GRT and FRE showed strong inhibition of CYP2C8 activity (7.69 ± 8.85 µg/mL and 8.93 ± 8.88 µg/mL, respectively) (Figure 3a). Both extracts moderately inhibited CYP3A4 activity (31.33 ± 4.69 µg/mL and 51.44 ± 4.31 µg/mL, respectively) (Figure 3b), while ASP displayed weak inhibition of CYP3A4 activity (69.57 ± 4.03 µg/mL) (Figure 3b).

### 2.4. Concentration-Dependent Screening of Compounds and Extracts

**Figure 4:**
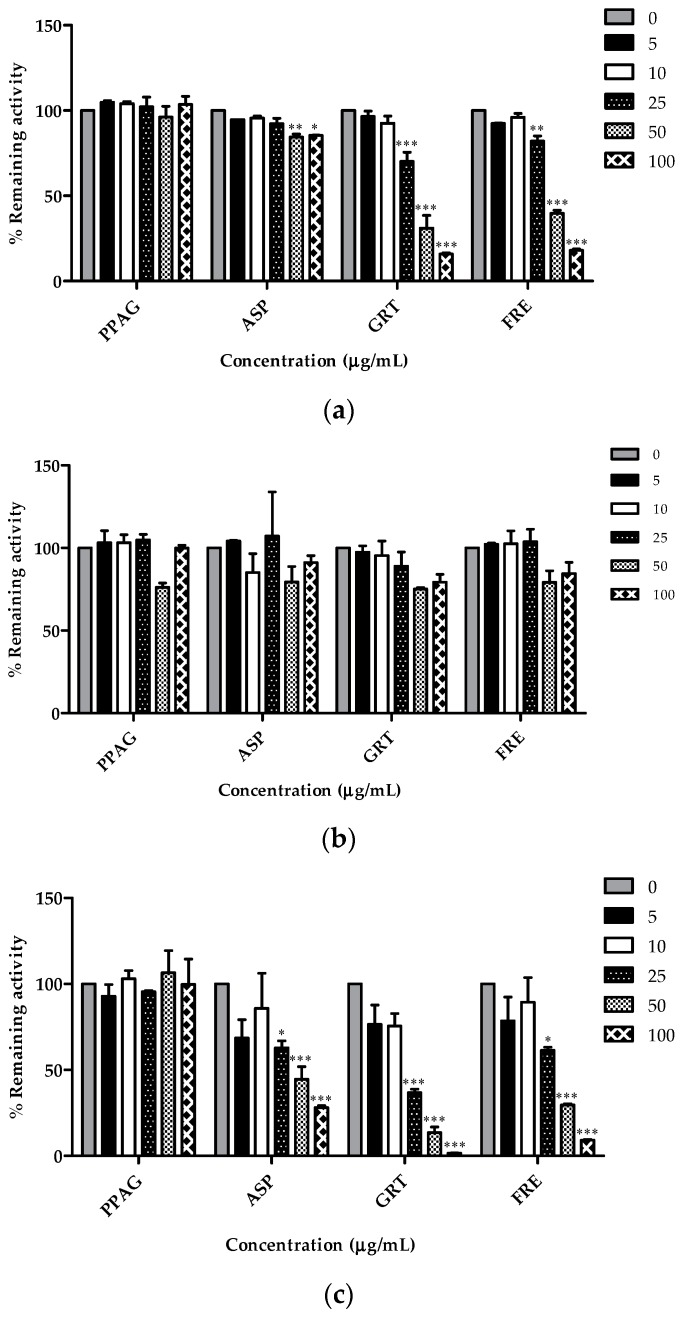
Percentage remaining activity of (**a**) CYP2C8; (**b**) CYP2C9 and (**c**) CYP3A4 following 30 min pre-incubation with PPAG (*Z*-2-(β-d-glucopyranosyloxy)-3-phenylpropenoic acid), ASP (aspalathin), GRT (unfermented rooibos extract), and FRE (fermented rooibos extract) at varying concentrations in the presence of NADPH with subsequent addition of substrates. Data are the average values of duplicate experiments with two replicates per sample (*n* = 4). * *p* < 0.05, ** *p* < 0.01, *** *p* < 0.001 when compared to other concentrations.

GRT and FRE reduced the remaining CYP2C8 activity in a moderate to strong concentration-dependent manner from 25 µg/mL (70.1% and 82.1%, respectively; *p* < 0.001), 50 µg/mL (31% and 39.7%, respectively; *p* < 0.001), and 100 µg/mL (15.9% and 18.1%, respectively; *p* < 0.001) (Figure 4a). ASP significantly inhibited CYP2C8 activity, albeit that the percentage remaining activity at 50 and 100 µg/mL was still at 84.4% and 85.5%, respectively. PPAG, ASP, GRT, and FRE did not significantly affect CYP2C9 enzyme activity (Figure 4b). ASP, GRT, and FRE reduced CYP3A4 activity at 25 µg/mL (62.9%, 36.9% and 61.4%, respectively; *p* < 0.001), 50 µg/mL (44.5%, 13.5% and 29.7%, respectively; *p* < 0.001), and 100 µg/mL (28.1%, 1.7% and 9.2%, respectively; *p* < 0.001) (Figure 4c).

### 2.5. Time-Dependent Screening of Compounds and Extracts on Enzyme Activity

**Figure 5:**
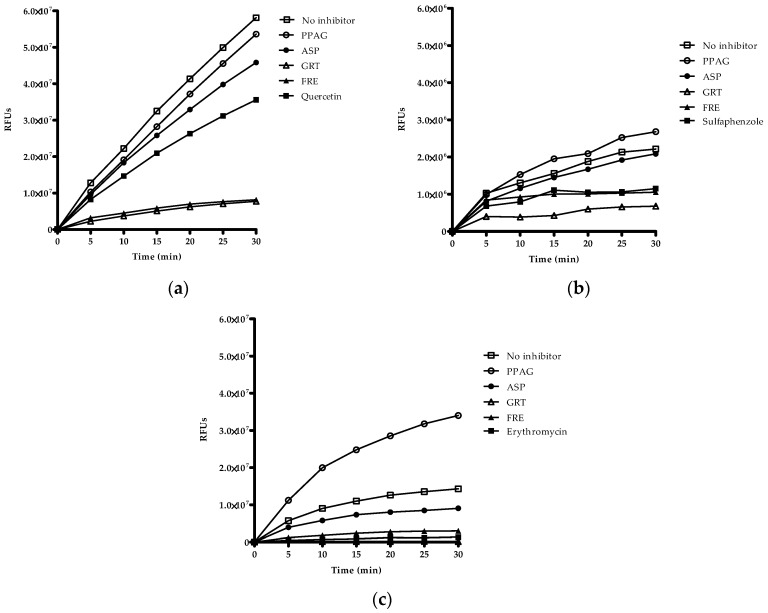
Screening of PPAG (*Z*-2-(β-d-glucopyranosyloxy)-3-phenylpropenoic acid), ASP (aspalathin), GRT (unfermented rooibos extract), and FRE (fermented rooibos extract) based on time-dependent inhibition of (**a**) CYP2C8 and (**b**) CYP2C9 at 100 µg/mL, and (**c**) CYP3A4 at 200 µg/mL, respectively, in the presence of NADPH for 30 min with subsequent addition of substrate. Quercetin, sulfaphenazole, and erythromycin at 10 µM were included as positive inhibitors. Data are presented as relative fluorescence units (RFU) for two independent assays done in duplicate (*n* = 4).

Time-dependent screening determines the inactivation of enzymes by the ligand or metabolites of the ligand generated over time. Both GRT and FRE showed time-dependent inhibition of CYP2C8 activity (Figure 5a). GRT showed a slight increase (*p* < 0.01) in inhibition of CYP2C9 activity after approximately 15 min, however, this inhibitory effect was more noticeable than for the positive inhibitor, sulfaphenazole (Figure 5b). PPAG demonstrated time-dependent inhibition (*p* < 0.05) of only CYP3A4 (Figure 5c). ASP indicated no time-dependent inhibitory activity. An interesting finding, however, is the time-dependent inhibition of CYP3A4 activity by GRT (*p* < 0.01) and FRE (*p* < 0.01), displaying a similar effect to that of erythromycin (Figure 5c).

## 3. Discussion

The prevalent use of natural products for the treatment of various medical conditions has increased the potential of medicinal herbs to interact with conventional drugs when consumed concomitantly [32,33]. Interactions between components of herbal medicines and drugs could alter the pharmacodynamics and pharmacokinetics of the latter, leading to adverse reactions and toxic effects or reduced drug efficacy [34,35,36,37,38].

Herbal extracts contain many constituents that can contribute to their effects at different concentrations [39]. These constituents include various bioactive compounds that can either activate or inhibit CYP3A4 [31]. *Echinacea purpurea*, a known plant-based product with constituents such as caffeic acid derivatives, amides, flavonoids, and glycosides, displayed weak inhibitory effects of CYP3A4 activity with IC_50_ values of 354–5394 µg/mL [39], and modestly induced hepatic CYP3A4 activity, thereby lowering the effective concentration of drugs, such as midazolam. For new chemical entities it is recommended that both reversible and time-dependent CYP inhibition is assessed to ascertain the risk of herb-drug or drug-drug interactions [40,41]. Therefore, in the current study, we screened for the inhibitory potential of the extracts and compounds using co-incubation (reversible inhibition) and pre-incubation (time-dependent inhibition) with CYP2C8, CYP2C9, and CYP3A4 Vivid^@^ recombinant enzymes.

Recent studies demonstrated the potential use of rooibos extracts [8,11] and compounds, such as ASP [19] and PPAG [24,25], as anti-diabetic and/or anti-obesity nutraceuticals. Plant extracts containing high levels of polyphenols are expected to have inhibitory effects on various CYPs including CYP2C8, CYP2C9, CYP2D6, and CYP3A4 [28,42]. Of these, CYP3A4 is commonly involved in herb-drug interactions as it metabolizes about 50% of clinically-prescribed medications [42,43,44]. Results obtained by Matsuda et al. [45] for rats ingesting rooibos tea for two weeks suggested a possible interaction between rooibos tea and medicines mediated by CYP3A. No details of the type of rooibos tea (unfermented or fermented) used to prepare the infusion for feeding to the rats were provided. For the present study, two extracts of rooibos, GRT and FRE, were tested for their ability to inhibit selected CYPs. GRT containing higher levels of polyphenols, in particular ASP (12.78% compared to 0.36%), demonstrated a more potent inhibition of CYP3A4 activity than FRE with IC_50_ values of 31.33 ± 4.69 µg/mL and 51.44 ± 4.31 µg/mL, respectively. ASP, as the major compound, moderately inhibited CYP3A4 activity with an IC_50_ value of 69.57 ± 4.03 µg/mL. Luteolin, the aglycone of rooibos flavone glucosides, orientin and isoorientin, and quercetin, the aglycone of rooibos flavonol glycosides, quercetin-3-*O*-robinobioside, rutin, hyperoside, and isoquercitrin, have previously been shown to inhibit CYP3A4 [46,47,48,49]. Quercetin is a more effective inhibitor of CYP3A4 than its 3-*O*-rutinoside, rutin [50]. The inhibition of CYP3A4 was also demonstrated to be both dose- and time-dependent. Therefore, they could potentially interfere with the metabolism and alter the pharmacodynamics of drugs, such as atorvastatin, cyclosporine, felodopine, simvastatin, midazolam, erythromycin, and doxorubicin, known to be metabolized by CYP3A4 [29,30,51].

CYP2C9 plays a role in the oxidation of xenobiotic and endogenous compounds and is responsible for the metabolism of 15%–20% of drugs undergoing phase I metabolism [52,53]. CYP2C9 is also involved in the metabolic clearance of therapeutic drugs such as oral hypoglycemics (pioglitazone, glyburide, and tolbutamide), cyclooxygenase-2 anti-inflammatories (celecoxib, ibuprofen, and naproxen), and oral anti-coagulants (warfarin) [29,30,51,54]. Changes in metabolic activity caused by genetic variants in CYP2C9 play a major role in the pathogenesis caused by adverse drug reactions [55]. Patients with low enzyme activity are at risk of adverse drug reactions from these CYP2C9 substrates [56]. We were unable to demonstrate a concentration dependent inhibitory effect for the extracts and ASP. However, a partial time-dependent decrease in CYP2C9 activity was shown for GRT and FRE with GRT demonstrating stronger inhibitory potential than the competitive CYP2C9 inhibitor, sulfaphenazole. When time-dependent inhibition is the major mode of action, the inhibitory effect of such a compound will be more prolonged specifically when multiple dosing is administered, such as the case for chronic medication [57]. Rooibos extracts, but not ASP or PPAG, when taken concomitantly with drugs, such as sulfonylureas, and specifically glyburide, could result in an exaggerated pharmacodynamic effect, thereby causing an increased risk of hypoglycemia [58,59].

A strong concentration- and time-dependent inhibition of CYP2C8 activity was observed for both GRT and FRE. However, ASP and PPAG had no effect on CYP2C8. This suggests that other phytochemicals or synergistic interactions of constituents in the extract account for this inhibitory effect. For compounds with the same basic flavonoid structure, the difference in inhibitory effect is related to their hydrophobicity [46]. Molecules more soluble in water do not make good substrates for P450 enzymes. Both ASP and PPAG are water-soluble. Other structural features of flavonoids that are important for their effect on CYPs are hydroxyl substitution (number and position) and the presence of double bonds [60]. Luteolin, containing several hydroxyl groups on the A- and B-rings, inhibit metabolism of drugs such as midazolam, whereas tangeretin, also a flavone, but without free hydroxyl groups on the A- and B-ring (i.e., methoxylated), increases midazolam metabolism [46]. Clinically, this could lead to increased concentrations of TZDs present in the circulation, thereby potentially altering their therapeutic dose.

This study demonstrates that GRT and FRE inhibited CYP2C8 and CYP3A4 activity in a dose- and time-dependent manner, whilst inhibiting CYP2C9 activity in a time-dependent manner only. ASP could only be implicated in CYP3A4 inhibition. PPAG did not display any inhibitory activities. These results indicate that rooibos extracts may potentially cause herb-drug interactions when co-administered with substrates or drugs metabolized by these P450 enzymes. This will include chronic medications, such as hypoglycemics (TZDs and sulfonylureas) and hypolipidemics (atorvastatin and simvastatin). As part of the drug discovery pipeline, it is important to demonstrate the potential of new therapeutics to interact with CYPs at a pre-clinical stage to avoid withdrawal once it enters the market. Future research should identify possible phytoconstituents of rooibos likely implicated in the observed interactions.

## 4. Materials and Methods

### 4.1. Plant Extracts

A fermented rooibos extract (FRE), previously shown to have anti-diabetic properties [8,14], was used in this study. In addition, a pharmaceutical-grade unfermented rooibos extract, Afriplex GRT (GRT), with a high aspalathin (ca. 12%) content was also included in the study. HPLC-diode array detection (DAD) quantification of the individual flavonoid and PPAG content of GRT was performed according to the method of Beelders et al. [61], as previously used for FRE (Figure 1b) [8].

### 4.2. Pure Compounds

**Figure 6:**
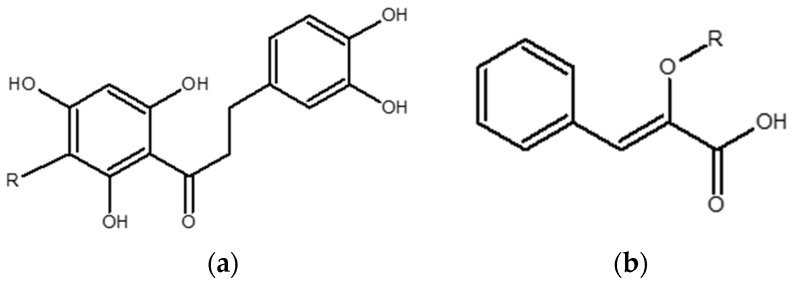
Structures of (**a**) aspalathin and (**b**) phenylpyruvic acid glucoside (PPAG) where R = β-d-glucopyranosyl.

*Z*-2-(β-d-glucopyranosyloxy)-3-phenylpropenoic acid (PPAG) (batch: MC1 (2)-248-91D) and aspalathin (ASP) (ca. 98%, batch SZI-356-54) were synthesized by High Force Research (Durham, UK) (Figure 6a,b).

### 4.3. Chemicals and Reagents

Ketoconazole, sulfaphenazole, erythromycin, quercetin, and solvents methanol, acetonitrile, and dimethyl sulfoxide (DMSO), were purchased from Sigma-Aldrich (St. Louis, MO, USA). Black Costar 96-well plates were obtained from Thermo Fischer Scientific (Pittsburgh, PA, USA). Vivid^®^ CYP2C8 Green Screening Kit with Vivid^®^ substrate, di-[benzyl-*O*-methyl]-fluorescein (DBOMF) and Vivid^®^ CYP3A4 and CYP2C9 Blue Screening Kits with Vivid^®^ substrate, 7-benzyl-oxymethyloxy-3-cyanocoumarin (BOMCC) were purchased from Life Technologies^™^ (Carlsbad, CA, USA). Purified water (double-distilled and deionized) was obtained from Millipore (Bedford, MA, USA).

### 4.4. Solvent Effect on CYPs

The effect of organic solvents such as methanol, acetonitrile and DMSO on Vivid^®^ recombinant CYP2C8, CYP2C9, and CYP3A4 assays was determined. A mixture containing 0.1% of organic solvent, reaction buffer (200 mM potassium phosphate (reaction buffer I) or 100 mM potassium phosphate (reaction buffer II)) and regeneration system (333 mM glucose-6-phosphate and 30 U/mL glucose-6-phosphate dehydrogenase in 100 mM potassium phosphate, pH 8.0) solution with the addition of each enzyme (CYP2C8, CYP2C9, or CYP3A4) was prepared. Two master mixes were prepared, namely Master Mix I, containing CYP (cytochrome) enzyme, regeneration system, and buffer, and Master Mix II, containing substrate (DBOMF/BOMCC), NADP^+^, and buffer. Master Mix I was added to extracts, compounds, and inhibitors and pre-warmed at 37 °C for 15 min. Thereafter, Master Mix II with NADP^+^ was also added to the extracts, compounds, and inhibitors to initiate the reaction and incubated at 37 °C for 30 min. Fluorescence was measured on a SpectraMax i3 plate reader (Molecular Devices, LLC, Sunnyvale, CA, USA) at 5 min intervals for 30 min to determine the reaction kinetics.

### 4.5. Qualitative Screening of Extracts and Compounds

Compounds, PPAG and ASP, and the rooibos extracts, GRT and FRE, were screened for their inhibitory effects on CYP2C8, CYP2C9, and CYP3A4 using one-point screening kinetics. Stock solutions (10 mg/mL) were prepared by diluting the extracts and compounds in distilled water. Briefly, inhibitors, extracts, and compounds were pre-incubated with either CYP2C8 or CYP2C9 at a concentration of 100 µg/mL, or CYP3A4 at a concentration of 200 µg/mL. In a black Costar 96-well plate, 3 µL of extracts or compounds (100 µg/mL) were added to 57 µL of reaction buffer. As per the manufacturer’s instruction, 50 µL of Vivid^®^ Master Pre-Mix (BACULOSOMES^®^ enzymes (CYP2C8, CYP2C9 or CYP3A4), regeneration system, reaction buffer (I/II), and NADP^+^) was added to each well. The plate was pre-incubated at 37 °C for 15 min. After incubation, 10 µL reconstituted DBOMF (CYP2C8) or BOMCC (CYP2C9 and CYP3A4) and NADP^+^ in Vivid^®^ reaction buffer I/II were added to each well and incubated for 30 min at 37 °C. Fluorescence was measured on a SpectraMax i3 plate reader at 5 min intervals to determine reaction kinetics. The reaction was stopped using cold 20% Tris base/80% acetonitrile. Enzyme activity was measured by formation of the metabolites at excitation and emission wavelengths of 485/530 nm (CYP2C8) and 406/460 nm (CYP2C9 and CYP3A4), respectively.

### 4.6. Quantitative Screening of Extracts and Compounds and IC_50_ Determination

A three-fold serial dilution of PPAG, ASP, GRT, and FRE was added to CYP2C8 and CYP2C9 (concentration range 100–0.41 µg/mL) and CYP3A4 (concentration range 200–0.82 µg/mL) to determine their respective IC_50_ concentrations. CYP2C8, CYP2C9, or CYP3A4 BACULOSOMES^®^ plus reagent and regeneration system in Vivid^®^ reaction buffer I/II added to a black Costar 96-well plate containing test extracts and compounds were incubated for 15 min at 37 °C. Thereafter, as previously described, the reaction was initiated by adding a mixture of reconstituted DBOMF (CYP2C8) or BOMCC (CYP2C9 and CYP3A4) and NADP^+^ in Vivid^®^ reaction buffer I/II and incubated for 30 min at 37 °C. After addition of the stop solution to terminate the reaction, fluorescence was determined at the relative excitation/emission wavelengths described in Section 4.5.

### 4.7. Time-Dependent Screening

Time-dependent inhibition (TDI) using the respective serial dilutions employed for IC_50_ determination (CYP2C8 and CYP2C9; 100–0.41 µg/mL and for CYP3A4; 200–0.82 µg/mL) was performed. The extracts and compounds were pre-incubated with CYP2C8, CYP2C9, or CYP3A4 BACULOSOMES^®^ and NADPH for 30 min. Thereafter, a mixture of substrate and NADP^+^ was added and the metabolite formation determined at 5 min intervals for 30 min at 37 °C using the SpectraMax i3 plate reader, as described in Section 4.5.

### 4.8. Concentration-Dependent Screening

Stock solutions (10 mg/mL) of PPAG, ASP, GRT, and FRE were prepared by dissolving the extracts and compounds in distilled water. Thereafter, 3 µL of extracts or compounds (100 µg/mL) were added to 57 µL of reaction buffer in a black Costar 96-well plate. The following concentrations, 100, 50, 25, 10, and 5 µg/mL were added to the plate in duplicate. Inhibition of CYP2C8, CYP2C9, and CYP3A4 BACULOSOMES^®^ was quantified, as described in Section 4.6.

### 4.9. Data Analysis

#### 4.9.1. Activity and IC_50_ Determination

The data generated were exported to an Excel (Microsoft Headquarters, One Microsoft Way, Redmond, WA, USA) worksheet and the amount of metabolite formed at various concentrations relative to the control was calculated using the following equation:

Relative percentage activity = 100 − (Test sample (extracts or compounds)/Average of positive control × 100)
(1)

The relative percentage activity was plotted against the log transformed concentrations of the extracts, compounds and positive controls, i.e. quercetin (positive CYP2C8 inhibitor), sulfaphenazole (positive CYP2C9 inhibitor), and ketoconazole (positive CYP3A4 inhibitor). A sigmoid curve was then fitted using a non-linear regression curve fit, dose response inhibition analysis was performed, and IC_50_ values were calculated using GraphPad Prism^®^ version 5.02 (GraphPad Software Inc., San Diego, CA, USA). IC_50_ values were calculated using the following equation:

Y = 100 − ((100 × (I)^H^)/ (IC_50_^H^ + (I)^H^)) (2)

where Y is the remaining enzyme activity (percentage control), (I), is the concentration of extracts and compounds, H is the Hill coefficient.

#### 4.9.2. Statistical Analysis

Data are means ± SEM. The percentage remaining activity was analysed using two-way ANOVA with *p* < 0.05 considered significant. Statistical analyses were performed using GraphPad Prism^®^ version 5.02 (GraphPad Software Inc.).

## 5. Conclusions

This in vitro study indicated that combining nutraceuticals containing rooibos extracts with drugs metabolized by CYP2C8 and CYP3A4 could potentially alter the pharmacodynamics and safety of these drugs. These findings still have to be confirmed in vivo.

## Supplementary Materials

Supplementary materials can be accessed at: http://www.mdpi.com/1420-3049/21/11/1515/s1.
