# Supplementary material for: Inhibitory Interactions of Aspalathus linearis (Rooibos) Extracts and Compounds, Aspalathin and Z-2-(β-d-Glucopyranosyloxy)-3-phenylpropenoic Acid, on Cytochromes Metabolizing Hypoglycemic and Hypolipidemic Drugs
Source: Molecules. 2016 Nov 12;21(11):1515. doi: 10.3390/molecules21111515 (PMC6273468; doi:10.3390/molecules21111515)
Supplement: Supplementary file 1 [file molecules-21-01515-s001.pdf]

Supplementary Materials: Inhibitory Interactions of *Aspalathus linearis* (Rooibos) Extracts and Compounds, Aspalathin and *Z*-2-(β-D-Glucopyranosyloxy)-3-phenylpropenoic Acid, on Cytochromes Metabolizing Hypoglycemic and Hypolipidemic Drugs

Oelfah Patel, Christo Muller, Elizabeth Joubert, Johan Louw, Bernd Rosenkranz and Charles Awortwe

Acetonitrile demonstrated an increase in fluorescence in CYP2C8, CYP2C9 and CYP3A4 activity over time (Figure S1a–c). Methanol showed an increase in fluorescence in both CYP2C8 and CYP3A4 (Figure S1a–c) activity and DMSO generated an increased effect in fluorescence of CYP2C8 (Figure S1c). These results showed that more fluorescence is generated than the control, suggesting that the solvents do not have an inhibitory effect on the respective enzymes. However, in CYP2C9 activity, DMSO and methanol showed a decrease in fluorescence over time (Figure S1b). Due to less fluorescence being generated than the control, the solvents may potentially have an inducing effect on the enzymes.

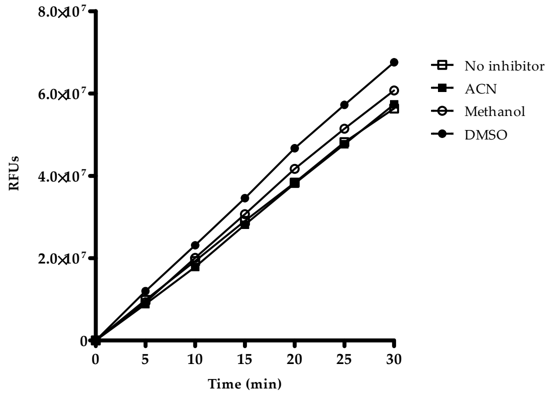

(**a**)

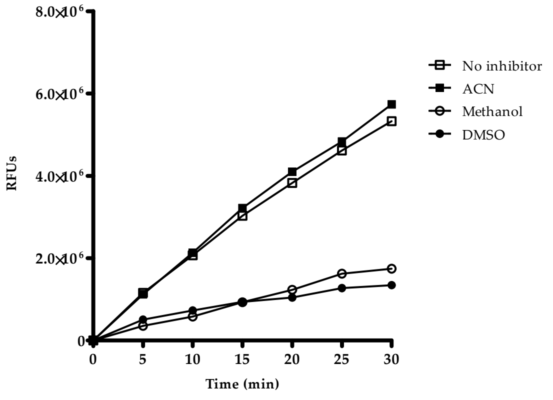

(**b**)

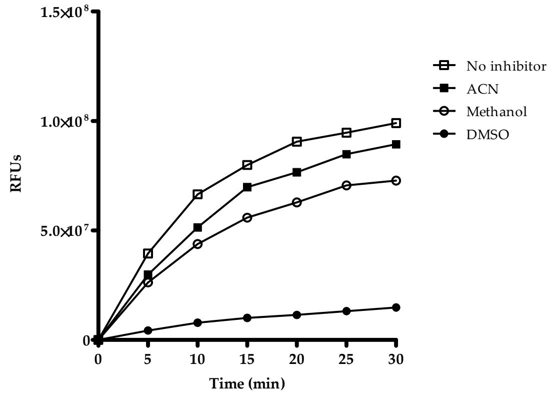

(**c**)

Figure S1. Solvent effect of DMSO (dimethyl sulfoxide), acetonitrile and methanol on (**a**) CYP2C8, (**b**) CYP2C9 and (**c**) CYP3A4 activity.
